# Impact of dose calculation accuracy during optimization on lung IMRT plan quality

**DOI:** 10.1120/jacmp.v16i1.5137

**Published:** 2015-01-08

**Authors:** Ying Li, Anna Rodrigues, Taoran Li, Lulin Yuan, Fang‐Fang Yin, Q. Jackie Wu

**Affiliations:** ^1^ Department of Radiation Oncology Duke University Medical Center Durham NC USA; ^2^ Department of Oncology The First Affiliated Hospital of Chongqing Medical University Chongqing China; ^3^ Medical Physics Graduate Program Duke University Medical Center Durham NC USA

**Keywords:** lung cancer, intensity‐modulated radiation therapy, dose calculation algorithm, optimization, treatment planning

## Abstract

The purpose of this study was to evaluate the effect of dose calculation accuracy and the use of an intermediate dose calculation step during the optimization of intensity‐modulated radiation therapy (IMRT) planning on the final plan quality for lung cancer patients. This study included replanning for 11 randomly selected free‐breathing lung IMRT plans. The original plans were optimized using a fast pencil beam convolution algorithm. After optimization, the final dose calculation was performed using the analytical anisotropic algorithm (AAA). The Varian Treatment Planning System (TPS) Eclipse v11, includes an option to perform intermediate dose calculation during optimization using the AAA. The new plans were created using this intermediate dose calculation during optimization with the same planning objectives and dose constraints as in the original plan. Differences in dosimetric parameters for the planning target volume (PTV) dose coverage, organs‐at‐risk (OARs) dose sparing, and the number of monitor units (MU) between the original and new plans were analyzed. Statistical significance was determined with a p‐value of less than 0.05. All plans were normalized to cover 95% of the PTV with the prescription dose. Compared with the original plans, the PTV in the new plans had on average a lower maximum dose (69.45 vs. 71.96 Gy, p=0.005), a better homogeneity index (HI) (0.08 vs. 0.12, p=0.002), and a better conformity index (CI) (0.69 vs. 0.59, p=0.003). In the new plans, lung sparing was increased as the volumes receiving 5, 10, and 30 Gy were reduced when compared to the original plans (40.39% vs. 42.73%, p=0.005; 28.93% vs. 30.40%, p=0.001; 14.11% vs. 14.84%, p=0.031). The volume receiving 20 Gy was not significantly lower (19.60% vs. 20.38%, p=0.052). Further, the mean dose to the lung was reduced in the new plans (11.55 vs. 12.12 Gy, p=0.024). For the esophagus, the mean dose, the maximum dose, and the volumes receiving 20 and 60 Gy were lower in the new plans than in the original plans (17.91 vs. 19.24 Gy, p=0.004; 57.32 vs. 59.81 Gy, p=0.020; 39.34% vs. 41.59%, p=0.097; 12.56% vs. 15.35%, p=0.101). For the heart, the mean dose, the maximum dose, and the volume receiving 40 Gy were also lower in new plans (11.07 vs. 12.04 Gy, p=0.007; 56.41 vs. 57.7 Gy, p=0.027; 7.16% vs. 9.37%, p=0.012). The maximum dose to the spinal cord in the new plans was significantly lower than in the original IMRT plans (29.1 vs. 31.39 Gy, p=0.014). Difference in MU between the IMRT plans was not significant (1216.90 vs. 1198.91, p=0.328). In comparison to the original plans, the number of iterations needed to meet the optimization objectives in the new plans was reduced by a factor of 2 (2–3 vs. 5–6 iterations). Further, optimization was 30% faster corresponding to an average time savings of 10–15 min for the reoptimized plans. Accuracy of the dose calculation algorithm during optimization has an impact on planning efficiency, as well as on the final plan dosimetric quality. For lung IMRT treatment planning, utilizing the intermediate dose calculation during optimization is feasible for dose homogeneity improvement of the PTV and for improvement of optimization efficiency.

PACS numbers: 87.55.D‐, 87.55.de, 87.55.dk

## I. INTRODUCTION

Current implementations of IMRT treatment planning with any commercial system utilize iterative optimization techniques, where the dose needs to be repeatedly calculated to assess the convergence to the optimization objectives. To improve the speed of such implementations, a fast and often simplified dose calculation algorithm is typically used during the iterative optimization process. At the conclusion of the optimization, a final dose calculation is performed using a more accurate algorithm to calculate the dose delivered to the patient. The trade‐off between efficiency and the accuracy of the simplified and accurate dose calculation algorithms is expressed in differences between the optimized and final dose distribution in accounting for tissue heterogeneity. This is particularly observable in the lung, where lateral electronic equilibrium between tissues of different densities breaks down under small field geometries.^1^, ^2^, ^3^

The fast dose calculation algorithm implemented during optimization in Eclipse utilizes a dose volume optimizer (DVO). The multiresolution dose calculation (MRDC) technique is also used within the DVO algorithm to further speed up the dose estimation and is based on the convolution–superposition principle. The final dose distribution is calculated using the more accurate analytical anisotropic algorithm (AAA), which has been shown to be superior in dose calculation for heterogeneous media and small fields.^4^, ^5^

Previous studies have investigated the variation in the final dose distribution of IMRT plans when fast dose calculation algorithms are used during the IMRT optimization process.^6^, ^7^ However, minimal information is available to understand the potential impact of the intermediate use of less accurate dose calculation algorithms on the quality and efficiency of the optimization results and the final plan quality. This effect could be more prominent when planning IMRT for lung cancers where the difference in electron density between air and water is substantial. Many dose calculation algorithms do not adequately predict dose in these situations. Using the fast dose calculation algorithms will potentially lead to false indications of achieving the constraints for the organs‐at‐risk (OARs) and planning target volume (PTV) during optimization and subsequently terminate the optimization prematurely. This dosimetric quality discrepancy between the plan calculated using a less accurate optimization algorithm and the plan calculated using a more accurate final dose calculation algorithm often results in a less‐than‐desirable final IMRT plan. Improvement of this discrepancy is challenging and could potentially be time‐consuming and labor‐intensive, since the optimization procedure must be repeated by, for example, changing constraints manually or adding optimization structures to achieve the desired dose distribution.

A new feature recently added to Eclipse v11 allows the calculation of an intermediate dose distribution during optimization using the AAA. However, quantifying improvements from this intermediate dose calculation utilizing AAA in comparison to using a standard fast dose calculation algorithm during optimization has not been previously reported. The purpose of this study, therefore, was to perform a comprehensive evaluation to understand the dosimetric impact of dose calculation algorithms used for the intermediate dose calculation on the final dosimetric quality of IMRT plans for lung tumors, in order to provide references for clinical physicists on the benefit of incorporating this module into the process of lung IMRT planning.

Dosimetric parameters and optimization efficiency for the PTV and OARs for IMRT plans with and without the intermediate dose calculation were compared.

## II. MATERIALS AND METHODS

### A. Treatment planning

Eleven free‐breathing clinically accepted IMRT treatment plans for lung tumors were randomly selected (i.e., different tumor volumes and locations) for this study. All patients underwent CT scanning for treatment planning. CTs were acquired from the mid‐neck to the mid‐abdomen level with slice thickness of 2.5 or 3 mm. The PTV was defined as an expansion by 3 to 5 mm from the internal target volume (ITV). The PTV location in the lung and volume for each patient is shown in Table 1.

All treatment plans utilized IMRT with 6 MV photon beams. Further, all plans used seven to nine beams, where the beam orientation was predominantly anterior–posterior, with both coplanar and noncoplanar beams, and fixed jaws. The prescription dose to the PTV was 64 Gy with 2 Gy/fraction. The objective of the inverse planning was to deliver the prescription dose to at least 95% of the PTV. The OARs included spinal cord, heart, esophagus, and total lung. In addition to general OAR sparing goals that follow the QUANTEC guidelines,^(8)^ physicians carefully examined patients’ anatomies and clinical indications and prescribed additional dose constraints on a case‐by‐case basis, seeking to maximize the OAR sparing.

**Table 1:** PTV location and volume for all patients. Right and left lung are denoted by RT and LT, while peripheral and midline locations are denoted by P and M

*Case No*.	*Location*		*PTV Volume (cm^3)^*
Patient 1	RT, M	Chest Wall	316.24
Patient 2	RT, M	Mediastinum	186.4
Patient 3	LT, M	Mediastinum	89.28
Patient 4	LT, M	Mediastinum	179.44
Patient 5	LT, M	Mediastinum	119.44
Patient 6	LT & RT, M	Mediastinum	262.73
Patient 7	LT, M	Mediastinum	205.71
Patient 8	RT, P	Upper Lobe	762.76
Patient 9	LT, M	Mediastinum	174.08
Patient 10	RT, P	Chest Wall	453.50
Patient 11	LT, M	Mediastinum & Chest Wall	214.32

### B. The optimization process

**Figure 1:**
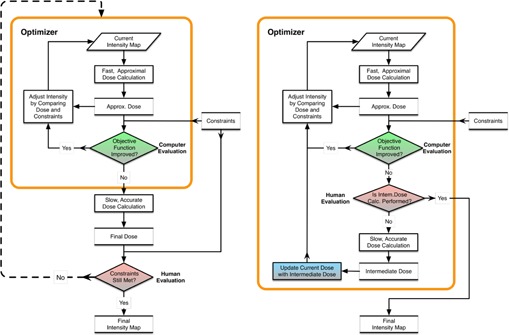
The IMRT optimization process for (left) the original plan without the intermediate dose calculation module and (right) the new plan with the intermediate dose calculation module. The orange box highlights the processes/operations performed within the optimizer.

The 11 clinically accepted treatments plans (”original” plans) were replanned (”new” plans) utilizing the intermediate dose calculation module. The optimization process, with and without the intermediate dose calculation module, is illustrated in Fig. 1. The main difference between these two methods of planning is the feedback of accurately calculated dose to the optimizer provided by the intermediate calculation module for the new planning process. For the original optimization (Fig. 1 (left)), the planner relies on a fast dose calculation algorithm (DVO) to perform dose calculation during the entire optimization process. The DVO algorithm is based on the convolution–superposition principle, and is calculated in a multiresolution fashion. Once the planner decides that the optimization has converged to the optimization objectives for the OARs and PTV (i.e., when the main objective function plateaus), the planner exits the optimization loop and performs the final dose calculation to evaluate the dose distribution. The final dose calculation is performed with the AAA, which is also a 3D pencil beam convolution superposition algorithm, but with much more accurate and complex modeling for the extrafocal photons, electron scattered from beam modifiers, and lateral scatter contributions.

Differences in the algorithms used during optimization and final dose calculation often lead to false indications of meeting PTV and OARs constraints. This discrepancy between the optimization algorithm perceived dosimetric quality and the actual dose distribution calculated by the final dose algorithm leads to additional optimization iterations to compensate for such discrepancies. This is depicted in Fig. 1 (left), where the optimized intensity and dose distribution must be adjusted (dashed outer loop). In this outer loop, the planner adjusts parameters during the repeat optimization process to make up the deficiencies for some specific dose‐volume objectives, using “ad hoc” techniques (e.g., adjusting optimization constraints and adding avoidance structures). When the optimization is completed, the final dose distribution is calculated again and, if not found to be satisfactory, the outer loop is repeated.

Figure 1 (right) shows the new optimization process implemented in the Eclipse treatment planning system v11 (Varian Medical Systems, Palo Alto, CA) with the new intermediate dose calculation module. In comparison to the original IMRT plan, an intermediate dose calculation during optimization with the DVO algorithm can be invoked. When the total objective function plateaus, the intermediate dose calculation is performed with the fluences achieved at the current stage of the optimization. The intermediate dose calculation uses the same AAA as in the final dose calculation of the IMRT plan. Once the intermediate dose calculation is completed, the dose distribution is compared with the internal, approximate dose distribution generated during the optimization. The difference between these two dose distributions is used for adjustment of the optimal fluences for the rest of the optimization. As a result of adjusting the fluences, the DVHs produced during optimization and final dose calculation are very similar. Therefore, the outer loop process (Fig. 1 (left)) is not needed in this new optimization process.

### C. Plan quality evaluation

DVHs were calculated for the PTV and OARs for the paired original and new IMRT plans for each patient.

For the PTV, the following metrics were compared between the paired treatment plans: Maximum dose ($D_{\text{max}}$, dose to 0.03 cc of PTV), homogeneity index (HI), and conformity index (CI). HI was defined as

(1) $$HI=\frac{D_{2}-D_{98}}{D_{T}}$$

in which $D_{2}$ and $D_{98}$ represent the dose to 2% and 98% of the target volume, respectively, and $D_{T}$ is the prescription dose.^(9)^ CI was defined as

(2) $$CI=\frac{PTV_{ref}}{V_{PTV}}\cdot \frac{PTV_{ref}}{V_{ref}}$$

where ${\text{PTV}}_{\text{ref}}$ represents the volume of the PTV that is covered by 95% of the prescription dose (60.8 Gy), $V_{\text{PTV}}$ is the planning target volume, and $V_{\text{ref}}$ represents the volume enclosed by 95% of the prescription dose.^10^, ^11^

For the OARs, spinal cord, heart, esophagus, and total lung were analyzed. The percent lung volume for different dose levels ($V_{5\text{Gy}},V_{10\text{Gy}},V_{20\text{Gy}},V_{30\text{Gy}}$), as well as the mean dose value ($D_{\text{mean}}$), were compared between the original and new IMRT plans. Select dose volume parameters for the esophagus ($V_{20\text{Gy}},V_{60\text{Gy}},D_{\text{mean}},D_{\text{max}}$), heart ($V_{40\text{Gy}},D_{\text{mean}},D_{\text{max}}$), and cord ($D_{\text{max}}$) were compared as well.

Finally, the number of MUs for each plan was compared to evaluate whether the additional optimization iterations in the optimization process impacted final MU number. Planning efficiency was evaluated by comparing the total time spent on the IMRT optimization between the original and new optimization processes, depicted in Fig. 1.

### D. Statistical analysis

Statistical analysis was performed using the SPSS statistical analysis software package (IBM Corp., Armonk, NY). The Wilcoxon signed‐rank test was performed for the average plan quality metrics derived from the original and new plans to infer statistical significance with a significance level (p‐value) set to 0.05.

## III. RESULTS

### A.1 Dose distribution

**Figure 2:**
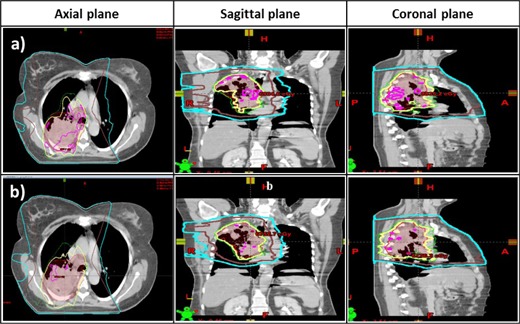
Final dose distribution for a free‐breathing IMRT plan (Patient #1) for a lung tumor superimposed on CT images in the axial, sagittal, and coronal plane for (a) the original plan without the intermediate dose calculation module, and for (b) the new plan with the intermediate dose calculation module. Isodose lines for 105% (purple), 100% (yellow), and 95% (green) of the prescription dose and 10 Gy (brown), and 5 Gy (cyan) are shown. Note the increased homogeneity in the target, which corresponds to a decrease in hot spots in the new plan.

Figure 2 shows a representative final dose distribution in the axial, sagittal, and coronal plane (Patient #1) for the (a) original plan and (b) new plan, to illustrate the effect of the intermediate dose calculation module on PTV coverage and OAR sparing. A noticeable difference is that the volume within the 105% isodose line (67.2 Gy) (pink) in the original plan is reduced in the new plan, which covers smaller and more scattered regions in the PTV. The 10 Gy and 5 Gy isodose lines are standard critical dose levels that physicians use to evaluate the quality of lung sparing. For the OARs, the 5 Gy isodose line (cyan) in the new plan is slightly tighter toward the PTV. These differences, however, do not significantly alter the overall dose distribution patterns in the OARs.

### A.2 Comparison between the original and new plans for the PTV

**Figure 3:**
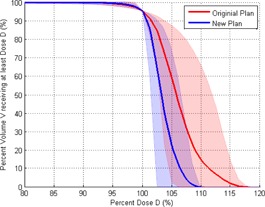
The average PTV DVH between the original (red) and new (blue) plans. The shaded area for both DVHs represents the range of all 11 plans. The PTV DVH for the new plan displays improved dose homogeneity and reduction in hot spots.

Figure 3 summarizes the DVH characteristics of the 11 patients by showing the average DVH comparison between the original and the new IMRT plans. The DVH of the PTV of the new plans displays a steeper gradient beyond the prescription dose compared to those from the original plans, corresponding to a reduction in the hot spots.

For the PTV, lower average $D_{2}$ (67.70±1.14 vs. 70.34±2.27Gy,p=0.002) and higher average $D_{98}$ (62.79±0.55 vs. 62.34±0.81Gy,p=0.01) were observed in the new plans when compared with the original plans. Further, statistical analysis showed that the HI was significantly better (0.08±0.03 vs. 0.12±0.04,p=0.002) for the new plans. Reduction of the average $D_{\text{max}}$ for the PTV was found for the new plans (69.45±1.73 vs. 71.96±2.36Gy,p=0.005). For the CI, the new plans were found to be perform better than the original plans (0.69±0.1 vs. 0.59±0.11,p=0.003). Comparison of the MUs, did not yield a significant difference between original and new plans (1216.9±332.4 vs. 1198.9±343.9,p=0.328). The results for the PTV plan quality metrics are summarized in Table 2.

**Table 2:** Summary of the DVH‐based analysis for PTV plan quality metrics and number of MUs represented as the average and standard deviation for 11 IMRT plans

*PTV*	*Original Plan*	*New Plan*	*Original ‐ New*	*p‐value*
$D_{2}$ (Gy)	70.34±2.27	67.70±1.14	2.65±1.68	0.002
$D_{98}$ (Gy)	62.34±0.81	62.79±0.55	‐0.45±0.48	0.010
$D_{\text{max}}$ (Gy)	71.96±2.36	69.45±1.73	2.51±1.68	0.005
HI	0.12±0.04	0.08±0.03	0.05±0.03	0.002
CI	0.59±0.11	0.69±0.10	‐0.09±0.07	0.003
MU	1198.91±343.93	1216.90±332.37	‐18±76.87	0.328

### B. Comparison between the original and new plans for the OARs

Overall, the OAR DVHs for the esophagus, heart, total lung, and spinal cord were similar between the original and new plans. Since the OARs varied in volume, the averaged OARs DVH washed out any subtle variation between the plans, thus not contributing to any new information. Therefore, only the previously mentioned dosimetric results for the OARs are summarized in Table 3.

For the esophagus, the mean dose, the maximum dose, and the volume receiving 20 and 60 Gy were lower in the new plans than in the original plans (17.91 vs. 19.24 Gy, p=0.004; 57.32 vs. 59.81 Gy, p=0.020; 39.34% vs. 41.59%, p=0.097; 12.56% vs. 15.35%, p=0.101). For the heart, the mean dose, the maximum dose, and the volume receiving 40 Gy were also lower in new plans (11.07 vs. 12.04 Gy, p=0.007; 56.41 vs. 57.7 Gy, p=0.027; 7.16% vs. 9.37%, p=0.012). For the total lung, the new plans had slightly lower volumes receiving doses of 5, 10, 20, and 30 Gy than the original plans (40.39% vs. 42.73%, p=0.005; 28.93% vs. 30.40%, p=0.001; 19.60% vs. 20.38%, p=0.052; 14.11% vs. 14.84%, p=0.031). The mean dose to the total lung was also lower (11.55 vs. 12.12 Gy, p=0.024). For the spinal cord, the max dose was lower in the new plans (29.1 vs. 31.39 Gy, p=0.014).

**Table 3:** Summary of the DVH‐based analysis for OARs in 11 IMRT plans. Metrics that show statistical significance are bolded

*OARs*		*Original Plan*	*New Paln*	*Original ‐ New*	*p‐value*
Esophagus	$V_{20}\;(\%)$	41.59±11.75	39.34±12.82	2.25±2.59	0.097
	$V_{60}\;(\%)$	15.35±9.43	12.56±9.16	2.79±2.11	0.101
	$D_{\text{mean}}\;(\text{Gy})$	19.24±10.32	17.91±10.07	1.32±1.14	**0.004**
	$D_{\text{max}}\;(\text{Gy})$	59.81±22.87	57.32±22.44	2.281±2.12	**0.020**
Heart	$V_{40}\;(\%)$	9.37±4.89	7.16±5.54	2.21±2.85	**0.012**
	$D_{\text{mean}}\;(\text{Gy})$	12.04±8.86	11.07±8.65	0.96±1.03	**0.007**
	$D_{\text{max}}\;(\text{Gy})$	57.70±27.18	56.41±26.64	1.29±1.79	**0.027**
Total lung	$V_{5}\;(\%)$	42.73±13.64	40.39±14.17	2.34±2.35	**0.005**
	$V_{10}\;(\%)$	30.40±9.21	28.93±9.64	1.47±1.44	**0.001**
	$V_{20}\;(\%)$	20.38±6.54	19.60±6.71	0.75±1.23	0.052
	$V_{30}\;(\%)$	14.84±5.32	14.11±5.43	0.74±0.98	**0.031**
	$D_{\text{mean}}\;(\text{Gy})$	12.12±3.61	11.55±3.53	0.58±0.69	**0.024**
Spinal Cord	$D_{\text{max}}\;(\text{Gy})$	31.39±9.71	29.1±10.49	2.38±2.25	**0.014**

### C. Planning efficiency evaluation

Due to the different dose calculation algorithms within the optimizer and outside the optimizer, the optimization process may need to be repeated several times to manually compensate for differences in the original plans. In the new plans, the number of optimization loop iterations to achieve a satisfactory final dose distribution was reduced from five to six to one to two due to the introduction of the intermediate dose calculation module. As a consequence, the total optimization time was reduced on average by 30%. For a clinical treatment planning time of 30–45 min, this translates to a reduction of approximately 10–15 min per case.

## IV. DISCUSSION

The choice of the dose calculation algorithm and how it is incorporated into the IMRT optimization affects the speed, accuracy, and optimality of the final dose distribution. More advanced dose calculation algorithms, such as the AAA, apply multiple photon kernels derived from Monte Carlo modeling to account for complex tissue heterogeneity inside the patient body. The accuracy of the AAA dose calculation has been verified with Monte Carlo simulations and experimental measurement with phantoms.^12^, ^13^, ^14^, ^15^, ^16^

However, in many treatment planning systems, a fast dose calculation algorithm is normally used to perform the repeated dose calculations during optimization to allow rapid completion of the optimization. Approximations and simplifications used by these fast dose calculation algorithms to achieve the dose calculation speed can result in final dose inaccuracies. Invoking an intermediate dose calculation using the AAA during the optimization is useful, especially if the DVH calculated during the optimization process deviates from the DVH produced from the final dose calculation. This often happens when density heterogeneities are present in the treated volume. This is in contrast to the original optimization process where “ad hoc” approaches, such as the use of optimization structures, are used to compensate for cold spots inside the PTV, and manual adjustment of parameters during the repeat optimization are often applied to mitigate the discrepancies between the optimization dose and final dose distributions. In this case, the “ad hoc” approach is needed, as the planner does not know the amount of adjustment needed to compensate for the differences caused by the dose calculation algorithms. The planner often focuses on achieving OAR results when using the “ad hoc” approach, which usually leads to relaxation of PTV goals and thus results in less favorable PTV dose distributions when compared with plans calculated with an intermediate dose calculation during optimization.

The results of this study showed that using the intermediate dose module during the optimization process improves both lung IMRT plan quality and planning efficiency. While it may be a foregone conclusion that the use of a more accurate dose calculation algorithm during optimization will lead to better plan quality, quantitative data on how much improvement can be achieved and its clinical impact are still important in order for clinical physicists to understand the impact of using this technique. As such, this study provides quantifiable benefits of using the intermediate dose calculation algorithm for lung IMRT treatment planning, and is thus of clinical importance, as it offers an objective evaluation of this feature and guidelines for centers that are currently not using this function or have been using it but are unsure of its actual benefit in terms of dosimetry and efficiency.

## V. CONCLUSIONS

This study represents the first systematic study of the effect of the intermediate dose calculation algorithm for lung IMRT treatment planning. In this study the benefit of using an intermediate dose calculation algorithm, which utilizes the AAA during optimization in a fluence‐based treatment planning system, was evaluated for lung IMRT treatment planning. Treatment plans generated with and without intermediate dose calculation were compared in terms of target and OAR dosimetry, as well as planning efficiency. The results indicated that for IMRT treatment planning of lung cancer, utilizing the intermediate dose calculation during optimization is feasible for dose homogeneity improvement and reduction of the maximum dose to the PTV, while substantially reducing the treatment planning time. OAR dose reductions were small, but still statistically significant. No significant changes were seen in the total number of MUs per plan. Optimization using the intermediate dose calculation for lung IMRT has, thus, been chosen as the default planning procedure in our clinic.
